# Tarsal tunnel syndrome caused by posterior facet talocalcaneal coalition: A case report

**DOI:** 10.1097/MD.0000000000020893

**Published:** 2020-06-26

**Authors:** Chang Hwa Hong, Hong Seop Lee, Won Seok Lee, Hyun Kwon Kim, Sung Hun Won, Eui Dong Yeo, Ki Jin Jung, Aeli Ryu, Jin Ku Kang, Dhong Won Lee, Woo Jong Kim

**Affiliations:** a Department of Orthopaedic Surgery, Soonchunhyang University Hospital Cheonan, Dongam-gu, Cheonan; b Department of Foot and Ankle Surgery, Nowon Eulji Medical Center, Eulji University, Nowon-gu; c Department of Orthopaedic Surgery, Soonchunhyang University Hospital Seoul, Yongsan-gu, Seoul; d Department of Orthopaedic Surgery, Soonchunhyang University Hospital Bucheon, Wonmi-gu, Bucheon; e Department of Orthopaedic Surgery, Veterans Health Service Medical Center, Seoul; f Department of Obsterics and Gynecology, Soonchunhyang University Hospital Cheonan, Dongnam-gu, Cheonan; g Department of Anesthesiology and Pain Medicine, Soonchunhyang University Hospital Cheonan, Dongam-gu, Cheonan; h Department of Orthopaedic Surgery, Konkuk University Medical Center, Gwangjin-gu, Seoul, Korea.

**Keywords:** posterior facet, talocalcaneal coalition, tarsal tunnel syndrome

## Abstract

**Rationale:**

Tarsal tunnel syndrome (TTS) is a compressive neuropathy of the posterior tibial nerve and its branches. Tarsal coalition is defined as a fibrous, cartilaginous, or osseous bridging of 2 or more tarsal bones. TTS with tarsal coalition is uncommon. Here, we present a rare example of successful surgical management of TTS with posterior facet talocalcaneal coalition.

**Patient concerns:**

A 74-year-old woman presented with hypoesthesia, numbness, and an intermittent tingling sensation on the plantar area over the right forefoot to the middle foot area. The hypoesthesia and paresthesia of the right foot began 6 years previously and were severe along the lateral plantar aspect. The symptoms were mild at rest and increased during daily activities. Tinel sign was positive along the posteroinferior aspect of the medial malleolus.

**Diagnosis:**

Lateral ankle radiography showed joint-space narrowing and sclerotic bony changes with a deformed C-sign and humpback sign. Oblique coronal and sagittal computed tomography revealed an irregular medial posterior facet, partial coalition, narrowing, and subcortical cyst formation of the posterior subtalar joint. Magnetic resonance imaging showed an abnormal posterior talocalcaneal coalition compressing the posterior tibia nerve. Electromyography and nerve conduction velocity studies were performed, and the findings indicated that there was an incomplete lesion of the right plantar nerve, especially of the lateral plantar nerve, around the ankle level.

**Interventions:**

Surgical decompression was performed. Intraoperatively, the lateral plantar nerve exhibited fibrotic changes and tightening below the posterior facet talocalcaneal coalition. The coalition was excised, and the lateral plantar nerve was released with soft-tissue dissection.

**Outcomes:**

The patient's symptoms of tingling sensation and hypoesthesia were almost relieved at 4 months postoperatively, but she complained of paresthesia with an itching sensation when the skin of the plantar area was touched. The paresthesia had disappeared almost completely at 8 months after surgery. She had no recurrence of symptoms at the 1-year follow-up.

**Lessons:**

The TTS with tarsal coalition is rare. Supportive history and physical examination are essential for diagnosis. Plain radiographs and computed tomography or magnetic resonance imaging are helpful to determine the cause of TTS and verify the tarsal coalition. After diagnosis, surgical excision of the coalition may be appropriate for management with a good outcome.

## 1 Introduction

Tarsal tunnel syndrome (TTS), first defined by Keck^[1]^ and Lam^[2]^ in 1962, is a compressive neuropathy of the posterior tibial nerve and its branches within the tarsal tunnel under the flexor retinaculum. Patients with TTS exhibit pain and dysesthesia in the distribution of the medial and lateral plantar nerves and may even exhibit sensory or motor changes. Various etiologies have been identified, including trauma, space-occupying lesions, foot deformities, and systemic disease.^[3–5]^

Tarsal coalition is a fibrous, cartilaginous, or osseous bridging of 2 or more tarsal bones, which has an incidence rate in the general population of approximately 1%.^[6,7]^ Talocalcaneal (TC) and calcaneonavicular (CN) coalition are common types of tarsal coalition.^[7,8]^ Tarsal coalition may cause TTS by presenting as a space-occupying lesion, although this is rare.^[9]^ Here, we present a case of successful treatment of TTS due to posterior TC coalition.

## 2 Case description

This case report was approved by the Institutional Review Board of Soonchunhyang University Hospital, Seoul, South Korea (IRB no: 2019-04-003). The patient provided consent for the publication of this report and the accompanying images.

**Figure 1:**
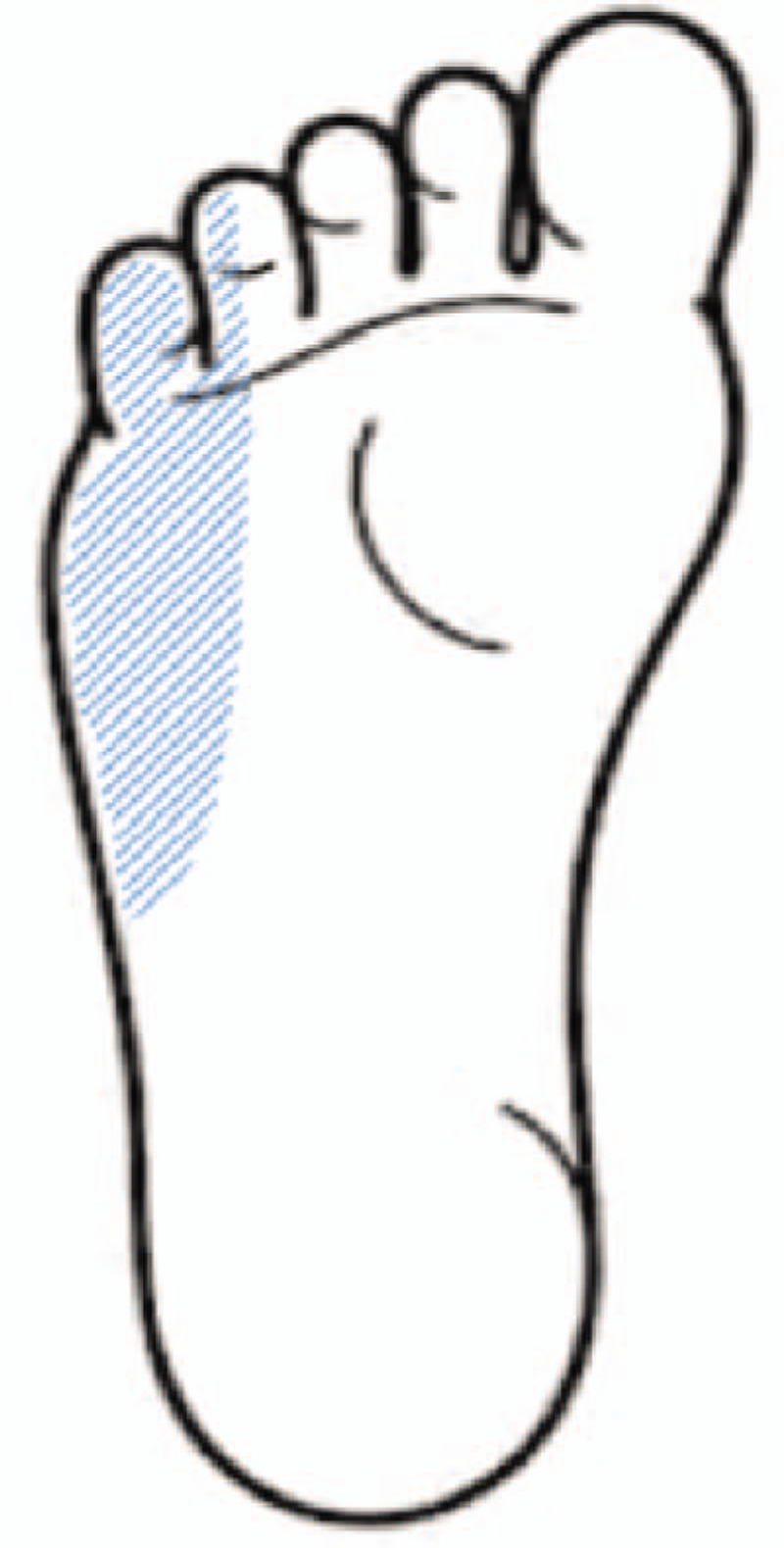
Distribution of hypoesthesia and paresthesia in our patient.

**Figure 2:**
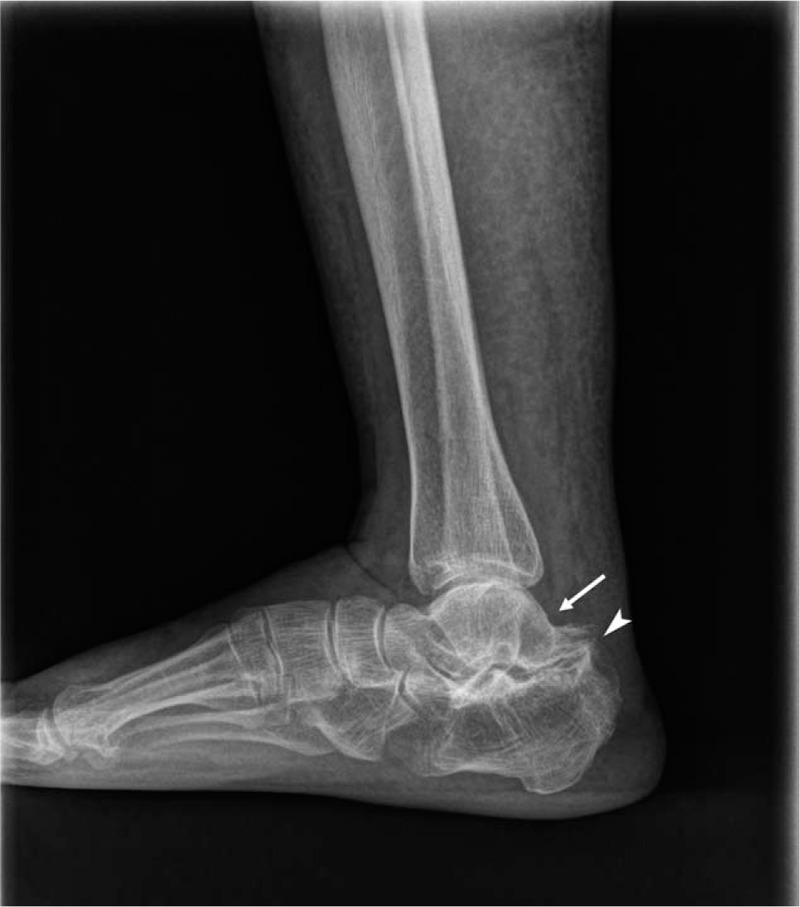
Preoperative plain standing lateral radiography view of the right ankle showing subtalar joint space narrowing and sclerotic bony changes with a deformed C-sign (arrow) and humpback sign (arrowhead).

**Figure 3:**
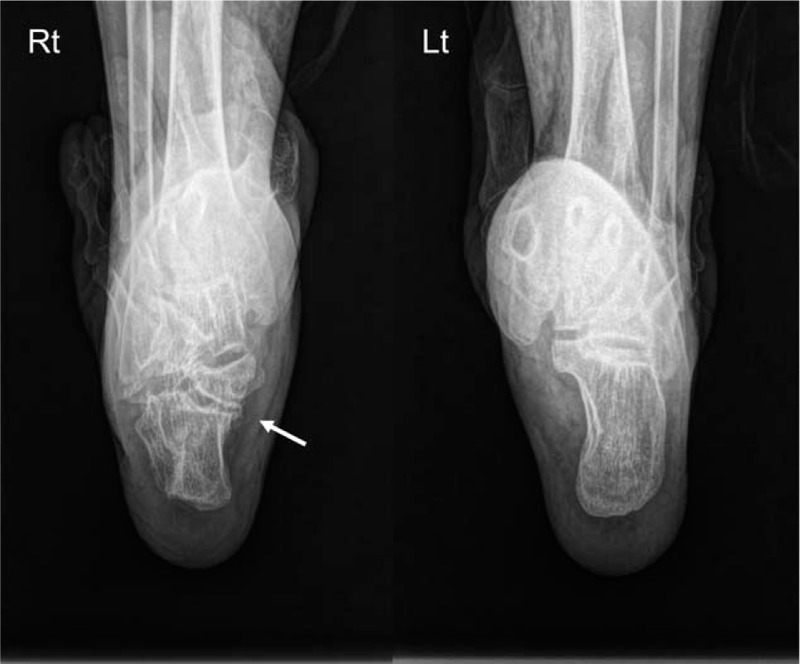
The joint space (arrow) of the medial posterior facet was narrowed, sclerotic, and irregular in both right (Rt) and left (Lt) visualizations using the Harris view.

**Figure 4:**
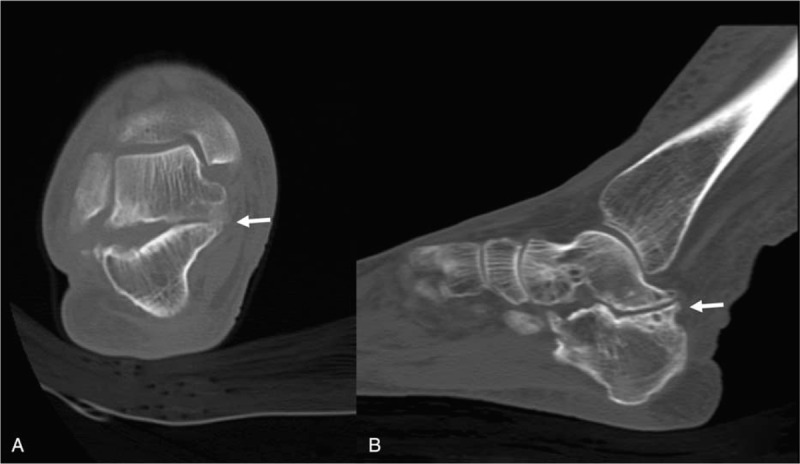
Computed tomography (CT) scan of the right ankle. Oblique coronal CT (A) and sagittal CT (B) showed irregular medial posterior facet, partial coalition, narrowing, and subcortical cyst formation at the posterior subtalar joint (arrow).

**Figure 5:**
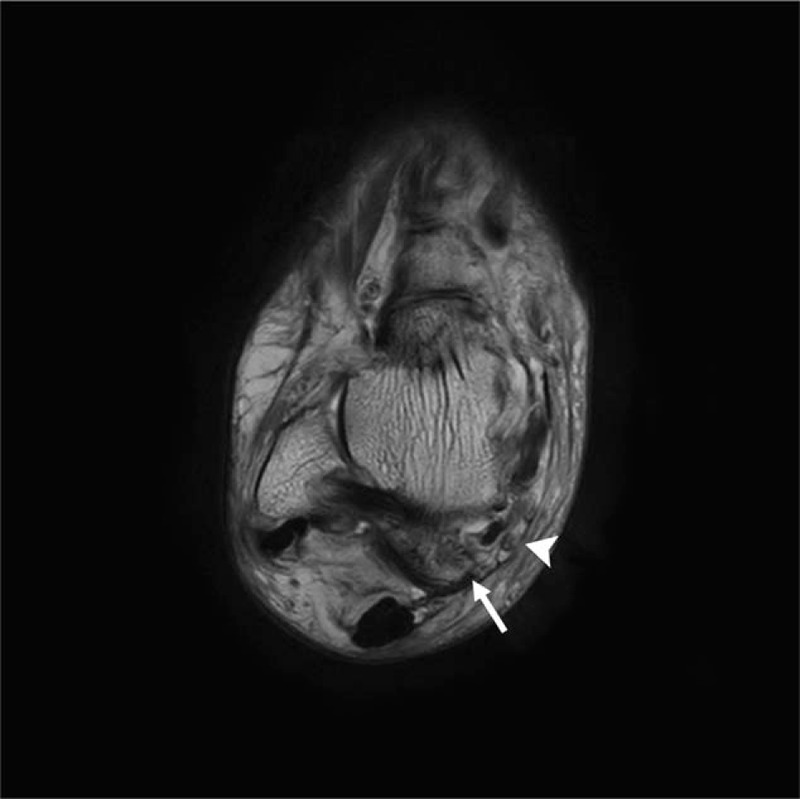
Preoperative axial T1-weighted magnetic resonance image of the right ankle showing an abnormal posterior talocalcaneal coalition (arrow) compressing the posterior tibial nerve (arrowhead).

A 74-year-old woman presented with hypoesthesia, numbness, and an intermittent tingling sensation on the plantar area over the right forefoot to the middle foot area. Although her childhood memories were not clear, she remembered her mother telling her that a condition resulting in pus from the lateral side of the right subtalar area had been cured when she was a child. In addition, a small scar was observed in this area. She first noticed hypoesthesia and paresthesia of the right foot 6 years ago, and these symptoms were severe along the lateral plantar aspect of the sore on the foot (Fig. 1). The symptoms were mild at rest and increased during daily activities. On initial physical examination, Tinel sign was positive along the posteroinferior aspect of the medial malleolus, but no palpable mass or tenderness was detected. A lateral ankle radiograph was obtained, which showed narrowing of the subtalar joint space and sclerotic bony changes with a deformed C-sign and humpback sign (Fig. 2). The joint space of the medial posterior facet was narrowed, sclerotic, and irregular when visualized using the Harris view (Fig. 3). On anteroposterior radiographs, the duck-face sign was not observed. Computed tomography (CT) and magnetic resonance imaging (MRI) were performed. Irregular medial posterior facet, partial coalition, narrowing, and subcortical cyst formation of the posterior subtalar joint were observed using oblique coronal CT and sagittal CT scans (Fig. 4). An abnormal posterior TC coalition compressing the posterior tibia nerve was observed using MRI (Fig. 5). Electromyography and a nerve conduction velocity study were performed, and the findings were compatible with an incomplete lesion of the right plantar nerve, especially of the lateral plantar nerve, around the ankle level. Therefore, we planned surgical treatment with a diagnosis of TTS caused by compression of the lateral plantar nerve by posterior facet coalition. The American Orthopedic Foot and Ankle Society (AOFAS) Ankle-Hind foot score was 60 points.

**Figure 6:**
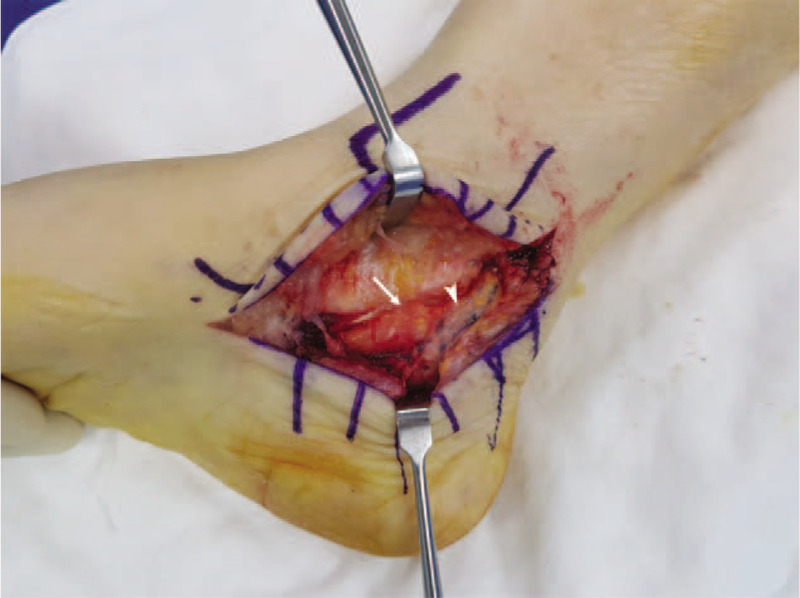
Intraoperative appearance of the posterior talocalcaneal coalition (arrow) compressing the lateral plantar nerve (arrowhead), which is a branch of the posterior tibial nerve.

**Figure 7:**
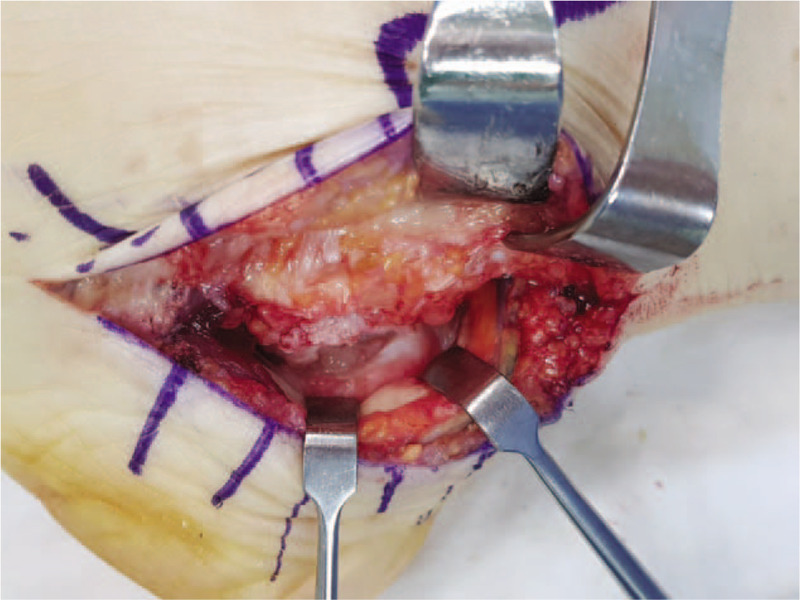
Excising the coalition relieved the nerve tension.

Surgical decompression was performed under general anesthesia with the patient placed in the supine position with a pneumatic tourniquet. Intraoperatively, the skin was incised for a length of 6 cm above the medial aspect of the right ankle. The lateral plantar nerve was found below the TC coalition (Fig. 6). Fibrotic change and tightening of the nerve were noted. Therefore, during excision of the coalition, we released the tension of the lateral plantar nerve with soft-tissue dissection (Fig. 7). At 4 months postoperatively, the patient's symptoms of tingling sensation and hypoesthesia were relieved almost completely, but she complained of paresthesia like an itching sensation when touching the skin of the plantar area. The paresthesia had disappeared almost completely at 8 months after surgery and the AOFAS Ankle-Hind Foot score improved to 88 points. She had no recurrence of symptoms at 1-year follow-up.

## 3 Discussion

In 1796, Buffon first described the tarsal coalition as a bony, cartilaginous, or fibrous connection between 2 or more tarsal bones.^[10,11]^ TC and CN types account for 90% of all tarsal coalitions observed at the posterior part of the foot,^[7,12]^ with rates of approximately 48.1% and 43.6%, respectively.^[7]^ The most typical location of a TC coalition is the middle subtalar facet, whereas a posterior subtalar facet coalition is extremely rare.^[13–16]^ However, the number of cases of TC coalition at the posterior subtalar facet is thought to be underestimated because of incorrect assessment of TC coalition.^[8,17]^ Taniguchi et al^[8]^ and Lee et al^[17]^ reported that the posterior subtalar facet has a higher frequency of TC coalition than the middle subtalar facet, suggesting that there may be ethnic differences between Western and Eastern populations.

Tarsal coalition can be completely asymptomatic and found incidentally on radiographic examinations. Vague pain in the subtalar joint can be aggravated by activity and relieved by rest in symptomatic patients. Pes planovalgus, reduced or absent subtalar motion, and peroneal muscle spasm, are also related to TC coalition. However, its combination with TTS is uncommon. As the symptoms of tarsal coalition have no distinctive features, plain radiography and additional imaging methods, such as CT or MRI, could be helpful in its diagnosis.^[17–20]^ As in our case, these methods could also be helpful for determining the location of the facet lesion, which is important when operating.

There have been few reports describing TTS, and none describing large numbers of cases.^[21,22]^ TTS caused by TC coalition has only been reported recently,^[21–24]^ and posterior subtalar facet TC coalition as a cause of TTS is extremely rare. In our case, the lateral plantar nerve was shown intraoperatively to be entrapped and tightened below the TC coalition. After excision of the coalition, the patient's symptom was relieved, suggesting that posterior subtalar facet TC coalition was the cause of TTS in this case.

The gold standard for treatment of tarsal coalition has yet to be determined. Coalition resection or arthrodesis is the primary surgical treatment option. There is also controversy regarding surgical treatment of TTS. However, a number of studies have shown that surgical release can be more effective than conservative treatment in TTS caused by space-occupying lesions. In our case, surgical excision of the coalition was a reliable technique to manage TTS with posterior facet TC coalition.

## 4 Conclusion

The TTS with posterior facet TC coalition is an extremely rare disease. Therefore, this possibility should be considered when taking the patient's history and performing physical examination. After diagnosis and preoperative planning, surgical decompression with excision of the coalition could be a good treatment option that leads to positive outcomes.

## Acknowledgment

The authors thank the Soonchunhyang University Research Fund (2019-0009) for its support.

## Author contributions

**Conceptualization:** Chang Hwa Hong, Eui Dong Yeo, Woo Jong Kim.

**Investigation:** Hyun Kwon Kim, Woo Jong Kim.

**Software:** Aeli Ryu.

**Supervision:** Hong Seop Lee, Sung Hun Won, Ki Jin Jung, Woo Jong Kim.

**Validation:** Dhong Won Lee.

**Visualization:** Won Seok Lee, Jin Ku Kang.

**Writing – original draft:** Won Seok Lee.

**Writing – review & editing:** Woo Jong Kim.
